# Does a Spruce Budworm Outbreak Affect the Growth Response of Black Spruce to a Subsequent Thinning?

**DOI:** 10.3389/fpls.2018.01061

**Published:** 2018-07-24

**Authors:** Sergio Rossi, Pierre-Yves Plourde, Cornelia Krause

**Affiliations:** ^1^Département des Sciences Fondamentales, Université du Québec à Chicoutimi, Chicoutimi, QC, Canada; ^2^Key Laboratory of Vegetation Restoration and Management of Degraded Ecosystems, Guangdong Provincial Key Laboratory of Applied Botany, South China Botanical Garden, Chinese Academy of Sciences, Guangzhou, China

**Keywords:** boreal forest, *Choristoneura fumiferana*, dendroecology, disturbance, growth rate, growth release, *Picea mariana*, sylviculture

## Abstract

In Canada, new forestry practices involving the natural dynamics of tree growth and regeneration are proposed for integrating forest management with biodiversity. In particular, the current spruce budworm [*Choristoneura fumiferana* (Clemens)] outbreak in northeastern North America is forcing natural resource managers to clarify the potential interactions between natural disturbances and commercial thinning. The aim of this study was to investigate if the spruce budworm outbreak of the 1970s affected the responses of black spruce [*Picea mariana* (Mill.) B.S.P.] to a subsequent thinning. Stem growth was reconstructed by measuring and cross-dating chronologies of tree-ring width of 1290 adult trees from 34 control and thinned stands within an area of 11,000 km^2^ in the boreal forest of the Saguenay-Lac-Saint-Jean region (QC, Canada). The treatment consisted of a low thinning performed during 1995–1999 that removed 25–35% of the basal area. Segmented models were applied to the tree-ring chronologies to define the growth pattern during the outbreak and thinning periods within a time window of 8 years, representing the average duration of the effects of defoliation on growth. Trees showed abrupt growth decreases during the outbreak, with the tree-ring index showing minimum values in 1977–1979. The tree-ring index had a flat trend before thinning, while it increased for 6–10 years after thinning. The growth pattern during the outbreak period was characterized by a reduction, mainly in trees with larger tree rings, while slow-growing trees showed less sensitivity to the disturbance. Thinning produced a significant increase in tree growth. No relationship was found between the effects of spruce budworm outbreaks in trees and the changes in growth pattern after thinning. If the timespan between the two disturbances exceeds 7 years, partial cutting can be applied independently of the growth reductions that had occurred during the outbreak. When applied in black spruce stands with high annual radial growth, thinning is expected to optimize the volume growth of the residual trees.

## Introduction

In North America, the cyclical stand renewal of several boreal species is closely related to two main disturbances: fire and insect outbreaks, the latter frequently being more important than the former (Gauthier et al., 2009). The larvae of spruce budworm [*Choristoneura fumiferana* (Clemens)], a native defoliator, feed on young and, more rarely, old needles consuming the photosynthetic tissues in spring and early summer (Gauthier et al., 2009). During outbreaks, the repeated defoliations dramatically deplete the reserves of trees, causing growth reductions and mortality of single trees or whole stands in a few years (Bouchard and Pothier, 2010). Although balsam fir [*Abies balsamea* (L.) Mill.] and white spruce [*Picea glauca* (Moench) Voss] are the main host species, recent studies have demonstrated the occurrence of distinct growth reductions in black spruce [*Picea mariana* (Mill.) B.S.P.] during the last four spruce budworm outbreaks (Gauthier et al., 2009; Tremblay et al., 2011). Because of its wide transcontinental distribution across the boreal forest of North America, black spruce is a keystone species of huge ecological and economic importance. Thus, all effects of natural disturbances on its growth can be spread over vast areas and have relevant consequences for the forest industry in northern regions.

The population of spruce budworm oscillates consistently over time, generating recurrent outbreaks (Montoro Girona et al., 2018). Jardon et al. (2003) demonstrated that the population increases in density at sub-continental scale every 25–38 years. The last outbreak occurred during 1970–1985 affecting a huge area with mature stands (Gauthier et al., 2009). The population of spruce budworm is now once again intensely rising. Within 4 years, the current outbreak has affected more than 4.2 M ha in Quebec (MFFP, 2017).

In Canada, the recent ecosystem-based management is attempting to modify conventional forestry with new practices closely adapted to the various regional differences and lower environmental impacts. This approach to silviculture is mainly based on the best available ecological knowledge to establish the most suitable harvesting strategies and targets (Gauthier et al., 2009). The application of these practices aims as far as possible to integrate management with biodiversity and the natural dynamics of growth and regeneration of the forest (Haeussler and Kneeshaw, 2003). For example, a number of partial cuttings have been developed to stimulate the growth of residual trees and advance their harvesting by simulating the forest gaps produced by the mortality following a spruce budworm attack (Zhang et al., 2006; Gauthier et al., 2009; Montoro Girona et al., 2016; Lemay et al., 2018).

Thinning is frequently used in even-aged stands of the boreal forest (Park and Wilson, 2007; Pelletier and Pitt, 2008). In ecosystems dominated by black spruce, canopy opening allows trees to receive more light for accomplishing their photosynthetic processes, warms up the soil, and increases plant nutrition by reactivating the cycling of nutrients accumulated in the thick organic layers (Pothier and Prevost, 2002; Vincent et al., 2009). However, because of the superficial distribution of the root system in black spruce, thinning could make stands more susceptible to wind, thus increasing the risk of windthrow (Cremer et al., 1982; Ruel et al., 2003; Gardiner et al., 2008). This expensive practice is worthwhile only if the reduction in stand density and competition allows the growth in diameter of the remaining trees to be substantially stimulated (Nyland, 2002; Vincent et al., 2009). Although encouraging results have been demonstrated for black spruce production (Vincent et al., 2009; Soucy et al., 2012; Pamerleau-Couture et al., 2015; Montoro Girona et al., 2017), some questions on the long-term effects of thinning on stand resistance and resilience remain unanswered (Gauthier et al., 2009; Bauce and Fuentealba, 2013). In particular, the current spruce budworm outbreak occurring in Eastern Canada is forcing forest scientists to urgently clarify the interactions between natural disturbances and silvicultural practices. The aim of this study was to investigate if the spruce budworm outbreak of the 1970s affected the responses of black spruce to a subsequent thinning realized in 1990s. To attain this objective, we reconstructed the stem growth by measuring and cross-dating tree-ring width in trees from 34 control and thinned stands.

## Materials and Methods

### Study Area

The study was conducted within an area of 1,1 M ha in the coniferous boreal forest of the Saguenay-Lac-Saint-Jean region (QC, Canada) belonging to the black spruce-feather moss domain. The region has a gently rolling topography with hills reaching 700 m a.s.l. on thick and undifferentiated glacial till deposits. The climate is boreal with cold winters and cool summers. The mean monthly temperatures can reach 17°C during July, but drop as far as −20°C in January. The mean annual temperature is approximately 2°C, with a May–September temperature of 13°C, and May–September rainfall of 401 mm. Winters are long, with daily mean temperatures around or below zero for a period of 180 days or more (Rossi et al., 2011).

### Stand Selection and Experimental Design

**FIGURE 1:**
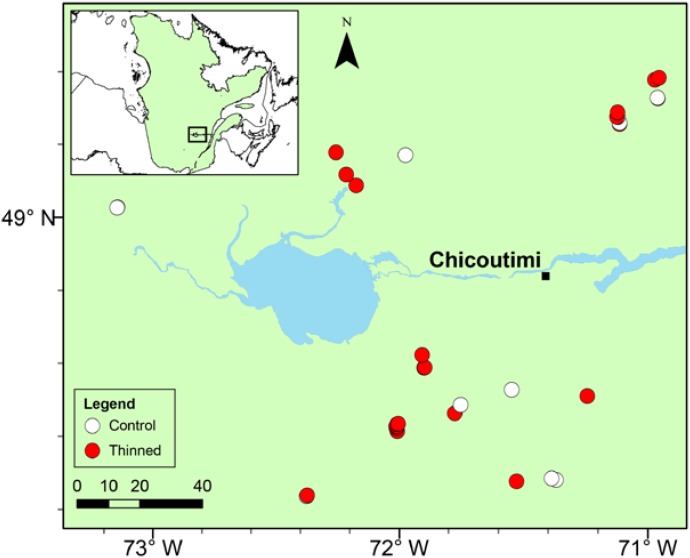
Location of the study stands in the Saguenay-Lac-Saint-Jean region. White and red dots correspond to control and thinned stands, respectively.

Thirty-four mature stands were selected (**Figure 1**). The stands, of fire origin, were even-aged and had a comparable species composition strongly dominated by black spruce, in association with balsam fir [*Abies balsamea* (L.) Mill.], Jack pine (*Pinus banksiana* Lamb.), white spruce [*Picea glauca* (Moench) Voss], white birch (*Betula papyrifera* Marsh.), and trembling aspen (*Populus tremuloides* Michx.). Twenty-three stands underwent a low thinning treatment during 1995–1999 (Supplementary Table S1). The thinning removed 25–35% of the stand basal area and was performed from extraction trails opened to establish an access network to the stands (Tremblay and Laflèche, 2012).

### Data Collection

In each stand, 20–84 dominant or co-dominant trees were selected (Supplementary Table S1). The large variability in the number of sampled trees was due to a preliminary analysis to assess the more suitable sample size, which was estimated to 35 trees (Vincent et al., 2009). In some cases, chronologies could not be cross-dated, which reduced the sample size to a value lower than 35. The trees had a height of between 9 and 18 m and a diameter at breast height (DBH) up to 20 cm. The decayed inner part of several trees prevented an exact evaluation of their age. On average, the minimal age of the trees was estimated to range between 56 and 126 years.

One increment core was collected with a Pressler borer 30 cm above the root collar of each tree and processed according to the standard methods in dendrochronology (Stokes and Smiley, 1968). The cores were collected close to the root collar to precisely estimate tree age and deepen as much as possible the tree-ring series. The samples were prepared according to the standard method in dendrochronology (Tremblay et al., 2011), and measured to the nearest 0.01 mm using either WinDENDRO (Regent Instruments Inc., Canada) or a manual Henson micrometer (LINTAB^TM^, Rinntech, Heidelberg, Germany). The ring-width series were corrected by visual cross-dating that was confirmed using the COFECHA computer program (Holmes, 1983). Each time series was standardized by applying a spline of 60 years to remove the long-term fluctuation. Autocorrelation in the data was not removed as the cumulative effect of defoliation was considered important for the analysis.

### Identification of the Growth Patterns

A segmented model was applied to the tree-ring chronologies to define the growth pattern during the outbreak and thinning periods. The model *E*(*x*) consisted of two linear segments according to the following system:

$$E(x)=\{\begin{array}{ll} a+bx, & x<X\\ c+dx, & x\geq X \end{array}$$

Where *x* represents the time axis, *a*, *b*, *c*, and *d* are the coefficients of the model, and *X* is the reference year for the outbreak [1978 (Krause et al., 2012)] or thinning (reported in **Table 1**). A condition was imposed that the two segments of the model were connected at their extremity in *X*:

a+bX=c+dX

**Table 1:** Effects of thinning (TH), growth rate (GR), and concavity during the spruce budworm outbreak (CO) on the growth pattern after thinning.

	GLM model		Binary logit model	
Source of variation	*F*-value	*P*	*F*-value	*P*
Thinning (TH)	51.71	<0.0001	15.71	<0.0001
Growth rate (GR)	4.16	<0.05	2.40	Ns
TH × GR	5.97	<0.05	0.35	Ns
CONCAVITY (CO)	0.01	Ns	0.62	Ns
CO × TH	0.09	Ns	0.37	Ns
CO × GR	0.14	Ns	0.17	Ns
CO × TH × GR	1.72	Ns	0.73	Ns

*The growth patterns tested by GLM and binary logit models are represented by the slope of the regression calculated for the four years after thinning and by the proportion of convex growth calculated for four years before and four years after thinning, respectively.*

Thus, the coefficients were related to the following relationship:

$$X=\frac{c-a}{b-d}$$

**FIGURE 2:**
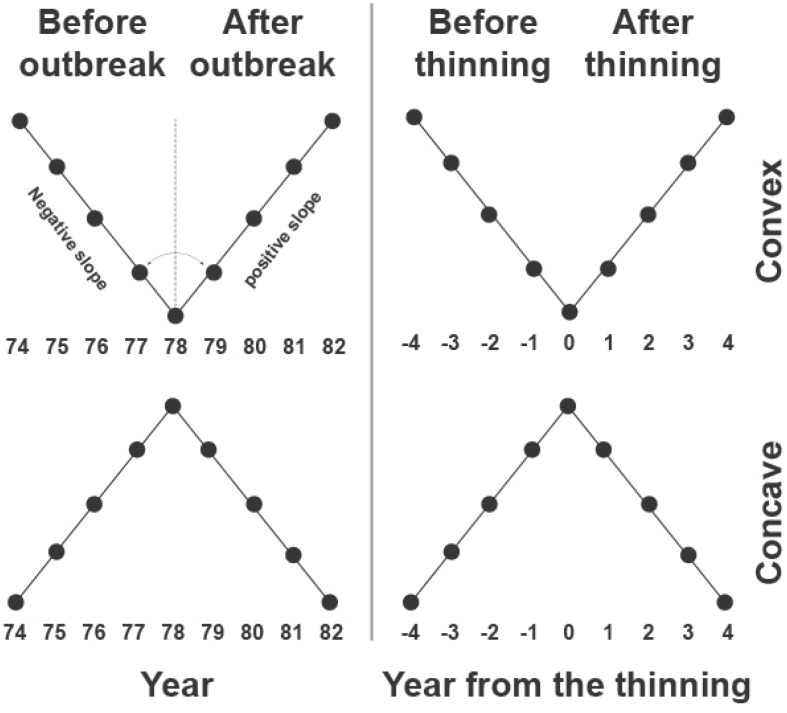
Example of the segmented models used for identifying convex and concave growth patterns in tree-ring index during outbreak and thinning.

The model was fitted to each tree-ring series and for the period of outbreak and thinning using the NLIN procedure in SAS 9.4 (SAS Institute Inc., Cary, NC, United States). A time window of 8 years (four before and four after outbreak or thinning) was used for the fitting, which was considered to represent the average duration of the effects of the outbreak on black spruce growth (Tremblay et al., 2011). The model for the outbreak period was fitted on standardized data after removing the long-term fluctuations assessed using a spline of 60 years. The segmented model identified two growth patterns according to the slopes *b* and *d* of the segments. A concave or convex pattern resulted if *b* > *d* or *b* < *d*, respectively, corresponding to a negative or positive growth after the event, as shown in the examples of **Figure 2**.

### Statistical Analyses

The trees were separated in two groups (slow- and fast-growing) of similar size according to their growth rates calculated as the average tree-ring width measured during the 6 years preceding the outbreak (1965–1970). The two resulting groups represented the trees with a growth rate lower and higher than the median, respectively. The effect of the growth pattern during the outbreak and growth rate on the slope of the regressions after thinning were assessed with a Generalized Linear Model (GLM). In addition, the binary responses to thinning, represented by the concave or convex growth patterns, were compared with the growth pattern during the outbreak and between the two growth rates using a binary logit model in SAS 9.4 (SAS Institute Inc., Cary, NC, United States).

## Results

### Tree-Ring Chronologies

**FIGURE 3:**
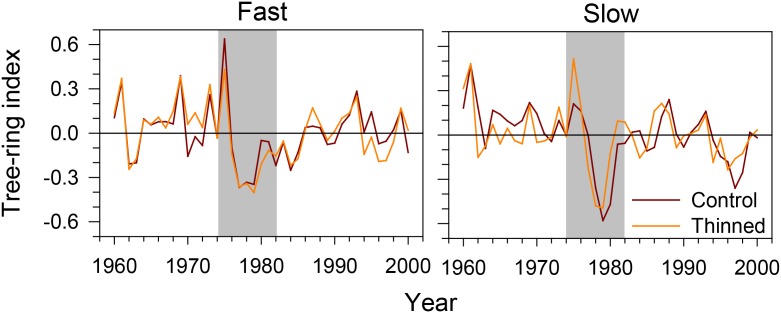
Tree-ring index of fast- and slow-growing black spruce trees during the spruce budworm outbreak in the Saguenay-Lac-Saint-Jean region (QC, Canada). Gray area represents the 8-year period considered by the successive analyses.

Both slow- and fast-growing trees showed an abrupt decrease in tree-ring width during the period of spruce budworm outbreak (**Figure 3**). The tree-ring index dropped dramatically in 1976, with the minimum values observed in 1977–1979. After that period, growth remained low for 2–3 years in slow-growing trees. A gradual recovery was observed in fast-growing trees. Control and thinned stands exhibited the same growth patterns, and seemed to respond similarly to the outbreak.

**FIGURE 4:**
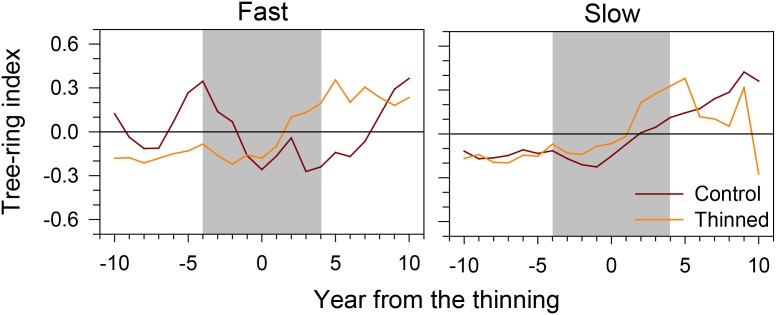
Tree-ring index of fast- and slow-growing trees before and after thinning. Gray area represents the 8-year period considered by the successive analyses.

During the 10 years before thinning, the tree-ring index had a flat trend, except for the fast-growing trees of control stands, which showed a more irregular pattern (**Figure 4**). Gradual increases in tree-ring width followed by a plateau were observed for 6–10 years after thinning. However, fast-growing trees of control stands increased their growth only 7 years after thinning.

### Regression Slopes

**FIGURE 5:**
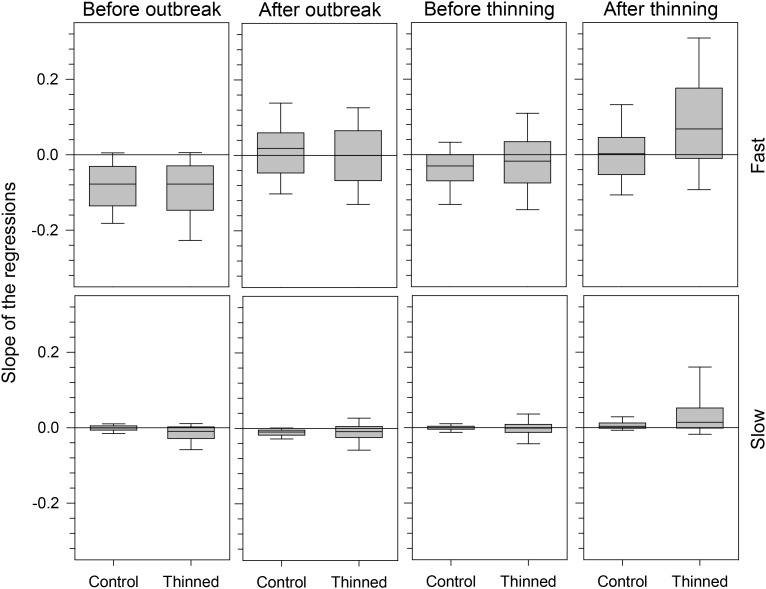
Slope of the regressions calculated during the four years before and after spruce budworm outbreak and thinning. Box plots represent the median, drawn as a horizontal solid line, with upper and lower quartiles and whiskers achieving the 10th and 90th percentiles.

The segmented models analyzed specifically the growth patterns occurring during the period close to the outbreak and thinning events and only in part reflected the results observed in the chronologies, which covered the average tree-ring width over a wider time window. Before 1978, i.e., the selected outbreak year, regressions had a decreasing pattern, showed by the negative values of their slopes, −0.8 × 10^−2^ and −9.4 × 10^−2^ on average for slow- and fast-growing trees, respectively (**Figure 5**). After the outbreak, regressions exhibited both negative and positive patterns except for slow-growing trees, which still had negative trends. Control and thinned stands showed similar growth patterns. Control and thinned stands had similar slopes before thinning, with values close to or below zero. After thinning, significant differences in slope were detected between treatments, with thinned stands having more positive regressions and slopes with higher absolute values than control stands (**Table 1**). A greater dispersion in the slope of the regressions occurred in fast-growing than in slow-growing trees, as also detected by significant results of GLM. No effect of the growth pattern occurring during the spruce budworm outbreak was observed in the slope of the regression after thinning (GLM, *p* > 0.05).

**FIGURE 6:**
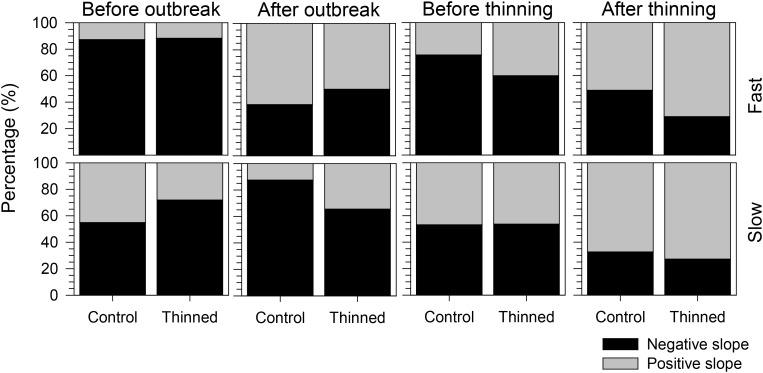
Proportion of positive and negative slopes of the regressions calculated during the 4 years before and after spruce budworm outbreak and thinning.

The proportion of negative and positive slopes of the regressions confirmed the previous results but clarified the main trends of the growth patterns (**Figure 6**). Both before and after the outbreak, negative slopes were more frequent than positive ones except for fast-growing trees after the outbreak. Negative slopes were observed before thinning, but greater proportions of positive slopes occurred afterward. Thinned stands had a higher percentage of positive slopes than control stands, but this difference was more marked for fast-growing trees.

### Growth Patterns

**FIGURE 7:**
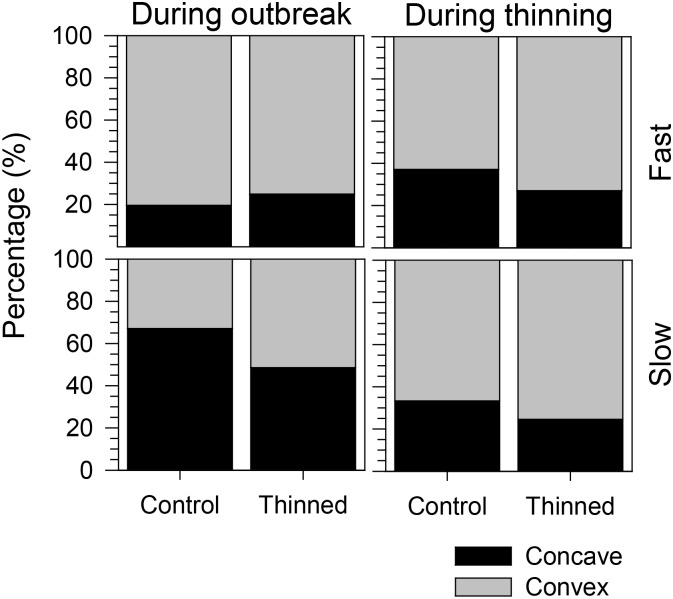
Proportions of concave and convex growth patterns during spruce budworm outbreak and thinning.

The growth pattern during the outbreak period was markedly convex in fast-growing trees (75 and 80% in thinned and control stands, respectively), while the slow-growing trees in control stands exhibited 67% of concave growth patterns (**Figure 7**). Convex growth patterns were observed during thinning in both control and thinned stands, although the proportion of convex growth patterns significantly increased in both slow- and fast-growing trees of thinned stands (GLM, *p* < 0.0001, **Table 1**). No significant difference was observed between slow- and fast-growing trees (GLM, *p* > 0.05). Similarly, GLM detected no effect of the growth patterns occurring during the spruce budworm outbreak on the concavity during thinning (GLM, *p* > 0.05).

## Discussion

### Thinning and Spruce Budworm Outbreak

Development and implementation of management strategies based on natural forest dynamics require the understanding of the ecosystem processes and their interactions with human activities (Gauthier et al., 2009). The effects of spruce budworm outbreak on stand dynamics in relationship with commercial thinning were previously approached by focussing on forest practices for reducing the damage produced by a successive outbreak of defoliators (Fuentealba and Bauce, 2012; Bauce and Fuentealba, 2013). A spruce budworm outbreak is currently producing dramatic growth reductions in trees that could also result in a diffuse mortality in several forested regions of northeastern North-America (Bouchard and Pothier, 2010). Such a mortality could also affect composition and structure of the forest stands on wide landscape scale. There is thus an urgent need to assess how the surviving stands respond to the silvicultural practices applied during the successive endemic period. In order to do this and for the first time to our knowledge, this study investigated the influence of a spruce budworm outbreak on the growth of trees submitted to a thinning treatment. Our analysis used tree-ring chronologies from 34 black spruce stands in the boreal forest of Quebec, Canada. The results indicated that the effect of thinning on tree-ring width of black spruce was independent of the growth reduction that trees had experienced during the outbreak. On the other hand, the growth rate of individual trees measured before the last spruce budworm outbreak was linked to the reduction in growth during the defoliation period, as well as the growth recovery following commercial thinning. Our findings detected less sensitivity to the disturbance in terms of tree-ring width reduction of slow-growing trees. This meta-analysis used data from a number of sites from a wide forest region, involving an important diversity in terms of stand density, composition, soil, and severity of defoliation. Consequently, we attempted to extract the general and common information contained in the growth time series despite the variability expected from such a heterogeneous dataset.

The timings of the treatment could explain the lack of interaction between outbreak and thinning. The treatment was performed between 1995 and 1999, 17–21 years after 1978, the reference date estimated by our chronologies as the peak of the outbreak period. Defoliated trees need some years to recover and we assume that a complete recovery is attained when individuals reach a growth rate similar to that exhibited before the outbreak (Morin, 1998). On average, our measurements estimated that a sustained growth was attained after 2–7 years, long before the thinning was done. During an outbreak, the root system is strongly affected, with abrupt growth reductions, absence of root growth, and mortality (Krause and Morin, 1995, 1999). The development of adventive roots is often observed to increase the efficiency of water uptake (Krause, 2006). Similarly, the photosynthetic needle biomass returns to a higher level and photosynthesis rate increases (Piene and MacLean, 1999). The allocation of carbohydrates to the cambium allows tree-rings to be formed as large as before the defoliation period or even larger (Pothier et al., 2005).

### Growth Reductions During Spruce Budworm Outbreak

Despite the marked growth reduction in the tree-ring chronologies during the outbreak, the growth response to the outbreak was not homogeneous among trees, as observed previously (Montoro Girona et al., 2017). Although most trees showed a clear contraction in growth, in some individuals the reduction in tree-ring width was marginal or lacking, confirming the literature (Vincent et al., 2009; Krause et al., 2012). Black spruce can be subjected to defoliation, but it is not the main host species of spruce budworm (Tremblay et al., 2011). It is likely that the different timings of larval emergence and bud break of black spruce prevent a continuous feeding by spruce budworm during the endemic periods and in part reduce the severity of the attacks during the outbreak (Hennigar et al., 2008; Colford-Gilks et al., 2012). Fuentealba and Bauce (2012) found a slower larval development on black spruce compared to the main host tree species. Black spruce is also known to develop smaller leaf biomass than the main host balsam fir (Nealis and Régnière, 2004). Pothier et al. (2012) observed that the growth reductions following moderate to severe defoliations were produced earlier in balsam fir than in black spruce. Moreover, the trees analyzed in this study were obviously only those that survived the outbreak, and which probably suffered less damage or better tolerated the effects of defoliation. The well-known capacity of adaptation and tolerance of black spruce to natural perturbations is also demonstrated by the low mortality after a spruce budworm outbreak (Colford-Gilks et al., 2012). This capacity of adaptation, in addition to the heterogeneous severity of the defoliation across stands could to some extent explain the variability observed in the growth reductions among trees.

The age of the trees plays an important role in the sensitivity of the stand to defoliation by spruce budworm, with mortality following spruce budworm outbreak increasing with stand age. Young individuals exhibit higher resistance than older trees to the damage caused by severe defoliations (Rossi et al., 2009a), because of their fast metabolism and greater capacity for enhancing photosynthesis (Boege, 2005; Mencuccini et al., 2005). According to our estimates, when the outbreak occurred in the studied sites, trees were between 30 and 40 years old, with the exception of two stands. This is only a minimum age calculated on complete cores. However, black spruce regenerates after fire within a few years, creating even-aged stands (Rossi et al., 2013). As a consequence, it is likely that trees in the same stand have the same or a similar age, thus, our estimate could be considered to approximate the real stand age.

### Growth Response to Thinning

Commercial thinning represent a practice alternative to the other traditional loggings with a lower impact on soil and canopy and are proposed as a suitable solution for maintain a heterogeneous pattern within and among stands (Gauthier et al., 2009). The effect of thinning on tree-ring width was expected, as previous studies have demonstrated the positive influence of the reduction in stand density and competition in improving black spruce growth (Soucy et al., 2012; Pamerleau-Couture et al., 2015; Lemay et al., 2017). Individuals with thinner tree-rings (called slow-growing trees), had a smaller and more homogeneous response to the treatment in terms of growth rate than trees with higher annual radial growth (fast-growing). Vincent et al. (2009) found that the response to thinning was related to stem diameter as well as competition, with smaller individuals having the highest growth increments after treatment. This was explained by the fact that small or suppressed individuals were more advantaged by the reduction in competition than dominant trees. However, Vincent et al. (2009) calculated a partial *R*^2^ of 0.042 for stem diameter, which was then supposed to affect only a minimal fraction of the growth response in trees. Thus, other, more specific approaches were proposed to disentangle and take into account the individual responses in growth within a stand (Montoro Girona et al., 2017).

The diameter was not related to the growth response after thinning in our analysis. On the other hand, growth rate did not correspond exactly to tree size. Growth rate changes with tree age: the classical growth trend is characterized by larger ring width close to the pith followed by a decrease in radial growth with age. This pattern is often not respected in forests with periodic spruce budworm outbreaks (Morin, 1998). The forest opening caused by tree mortality leads to gaps in the natural forest and the radial growth pattern can vary greatly between individuals (Rossi et al., 2009b; Montoro Girona et al., 2017). As spruce budworm has a cyclic recurrence every 30–35 years, it is possible that the defoliation periods in the 1950s had already determined a separation of trees into the two growth classes (slow- and fast-growing). Overall, the growth rate in black spruce before a thinning event seems to be closely connected to the successive growth release of trees. The problem of the individual variability and homogeneity in growth within the stands still remain partially unknown and require further and deeper investigations (Pamerleau-Couture et al., 2015; Montoro Girona et al., 2017).

## Conclusion

In this study, we measured and cross-dated tree-ring width in black spruce stands to answer the question if the spruce budworm outbreak of the 1970s affected the growth responses to the commercial thinning realized in 1990s. No relationship was found between spruce budworm outbreaks and changes in growth pattern after commercial thinning. If the timespan between the two disturbances is sufficient, more than 7 years, partial cutting can be applied without affecting the success of the growth release. However, the growth release after thinning seems to be related to the growth rate of trees, with the higher increases being concentrated in fast-growing individuals. Based on the results of this study, strategies of forest management should select black spruce stands with relatively high annual radial growth for thinning in order to optimize the volume growth of the residual trees.

## Author Contributions

CK designed the study. P-YP performed the field and lab work. SR analyzed the data and wrote the first draft. CK and P-YP prepared the final version.

## Conflict of Interest Statement

The authors declare that the research was conducted in the absence of any commercial or financial relationships that could be construed as a potential conflict of interest. The reviewer MG declared a past co-authorship with one of the authors SR to the handling Editor.

## References

[B1] Bauce É Fuentealba A. (2013). Interactions between stand thinning, site quality and host tree species on spruce budworm biological performance and host tree resistance over a 6 year period after thinning. *For. Ecol. Manage.* 304 212–223. 10.1016/j.foreco.2013.05.008

[B2] Boege K. (2005). Influence of plant ontogeny on compensation to leaf damage. *Am. J. Bot.* 92 1632–1640. 10.3732/ajb.92.10.1632 21646080

[B3] Bouchard A. Pothier D. (2010). Spatiotemporal variability in tree and stand mortality caused by spruce budworm outbreaks in eastern Quebec. *Can. J. For. Res.* 40 86–94. 10.1139/X09-178

[B4] Colford-Gilks A. K. Maclean D. A. Kershaw J. A. Béland M. (2012). Growth and mortality of balsam fir- and spruce-tolerant hardwood stands as influenced by stand characteristics and spruce budworm defoliation. *For. Ecol. Manag.* 280 82–92. 10.1016/j.foreco.2012.05.023

[B5] Cremer K. W. Borough C. J. Mckinnel F. H. Carter P. R. (1982). Effect of stocking and thinning on wind damage in plantations. *N. Z. J. For. Sci.* 12 244–268.

[B6] Fuentealba A. Bauce É (2012). Soil drainage class, host tree species, and thinning influence host tree resistance to the spruce budworm. *Can. J. For. Res.* 42 1771–1783. 10.1002/ps.2253 21796758

[B7] Gardiner B. Byrne K. Hale S. Kamimura K. Mitchell S. J. Peltola H. (2008). A review of mechanistic modelling of wind damage risk to forests. *For. Int. J. For. Res.* 81 447–463. 10.1093/forestry/cpn022

[B8] Gauthier S. Vaillancourt M.-A. Leduc A. De Grandpré L. Kneeshaw D. Morin H. (2009). *Ecosystem Management in the Boreal Forest.* Québec, QC: Presses de l’Université du Québec.

[B9] Haeussler S. Kneeshaw D. D. (2003). “Comparing forest management to natural processes,” in *Towards Sustainable Management*, eds Burton P. J. Messier C. Smith D. W. Adamowicz W. L. (Ottawa, ON: NRC Research Press), 307–368.

[B10] Hennigar C. R. Maclean D. A. Quiring D. T. Kershaw J. A. (2008). Differences in spruce budworm defoliation among balsam fir and white, red, and black spruce. *For. Sci.* 54 158–166.

[B11] Holmes R. L. (1983). Computer-assisted quality control in tree-ring dating measurement. *Tree Ring Bull.* 43 69–78.

[B12] Jardon Y. Morin H. Dutilleul P. (2003). Periodicity and synchronism of spruce budworm outbreaks in Quebec. *Can. J. Forest Res.* 33 1947–1961.

[B13] Krause C. (2006). Growth development of a balsam fir (*Abies balsamea* (L.) Mill.) origination from layering. *Dendrochronologia* 23 139–143. 10.1016/j.dendro.2005.12.001

[B14] Krause C. Luszczynski B. Morin H. Rossi S. Plourde P.-Y. (2012). Timing of growth reductions in black spruce stem and branches during the 1970s spruce budworm outbreak. *Can. J. For. Res.* 42 1220–1227. 10.1139/x2012-048

[B15] Krause C. Morin H. (1995). Changes in radial increment in stems and roots of balsam fir [*Abies balsamea* (L) Mill] after defoliation by spruce budworm. *For. Chron.* 71 747–754. 10.5558/tfc71747-6

[B16] Krause C. Morin H. (1999). Tree-ring patterns in stem and root systems of black spruce (*Picea mariana*) caused by spruce budworms. *Can. J. For. Res.* 29 1583–1591. 10.1139/x99-138

[B17] Lemay A. Krause C. Achim A. Bégin J. (2018). Growth and wood quality of black spruce and balsam fir following careful logging around small merchantable stems (CLASS) in the boreal forest of Quebec, Canada. *For. Int. J. For. Res.* 91 271–282. 10.1093/forestry/cpw060

[B18] Lemay A. Krause C. Rossi S. Achim A. (2017). Xylogenesis in stems and roots after thinning in the boreal forest of Quebec, Canada. *Tree Physiol.* 37 1554–1563. 10.1093/treephys/tpx082 28985379

[B19] Mencuccini M. Martínez-Vilalta J. Vanderklein D. Hamid H. A. Korakaki E. Lee S. (2005). Size-mediated ageing reduces vigour in trees. *Ecol. Lett.* 8 1183–1190. 10.1111/j.1461-0248.2005.00819.x 21352442

[B20] MFFP (2017). *Aires Infestées par la Tordeuse des Bourgeons de L’épinette au Québec en 2017-Version 1.0.* Québec: Direction de la Protection des Forêts, Ministère des Forêts, de la Faune et des Parcs.

[B21] Montoro Girona M. Morin H. Lussier J.-M. Walsh D. (2016). Radial growth response of black spruce stands ten years after experimental shelterwoods and seed-tree cuttings in boreal forest. *Forests* 7:240 10.3390/f7100240

[B22] Montoro Girona M. Navarro L. Morin H. (2018). A secret hidden in the sediments: lepidoptera scales. *Front. Ecol. Evol.* 6:2 10.3389/fevo.2018.00002

[B23] Montoro Girona M. Rossi S. Lussier J.-M. Walsh D. Morin H. (2017). Understanding tree growth responses after partial cuttings: a new approach. *PLoS One* 12:e0172653. 10.1371/journal.pone.0172653 28222200 PMC5319695

[B24] Morin H. (1998). Importance et évolution des épidémies de la tordeuse des bourgeons de l’épinette dans l’est du Canada: l’apport de la dendrochronologie. *Géogr. Phys. Quat.* 52 237–244. 10.7202/004856ar

[B25] Nealis V. G. Régnière J. R. (2004). Insect-host relationships influencing disturbance by the spruce budworm in a boreal mixedwood forest. *Can. J. For. Res.* 34 1870–1882. 10.1139/x04-061

[B26] Nyland R. D. (2002). *Silviculture: Concepts and Applications*. New York, NY: McGraw-Hill.

[B27] Pamerleau-Couture É Krause C. Pothier D. Weiskittel A. (2015). Effect of three partial cutting practices on stand structure and growth of residual black spruce trees in north-eastern Quebec. *Forestry* 88 471–483. 10.1093/forestry/cpv017

[B28] Park A. Wilson E. R. (2007). Beautiful plantations: can intensive silviculture help Canada to fulfill ecological and timber production objectives? *For. Chron.* 83 825–839. 10.5558/tfc83825-6

[B29] Pelletier G. Pitt D. G. (2008). Silvicultural responses of two spruce plantations to midrotation commercial thinning in New Brunswick. *Can. J. For. Res.* 38 851–867. 10.1139/X07-173

[B30] Piene H. MacLean D. A. (1999). Spruce budworm defoliation and growth loss in young balsam fir: patterns of shoot, needle and foliage weight production over a nine-year outbreak cycle. *For. Ecol. Manag.* 123 115–133. 10.1016/S0378-1127(99)00023-7

[B31] Pothier A. Elie J.-G. Auger I. Mailly D. Gaudreault M. (2012). Spruce budworm-caused mortality to balsam fir and black spruce in pure and mixed conifer stands. *For. Sci.* 58 24–33. 10.5849/forsci.10-110

[B32] Pothier D. Mailly D. Tremblay S. (2005). Predicting balsam fir growth reduction caused by spruce budworm using large-scale historical records of defoliation. *Ann. For. Sci.* 62 261–267. 10.1051/forest:2005018

[B33] Pothier D. Prevost M. (2002). Photosynthetic light response and growth analysis of competitive regeneration after partial cutting in a boreal mixed stand. *Trees* 16 365–373.

[B34] Rossi S. Morin H. Deslauriers A. (2011). Multi-scale influence of snowmelt on xylogenesis of black spruce. *Arct. Antarct. Alp. Res.* 43 457–464. 10.1657/1938-4246-43.3.457

[B35] Rossi S. Morin H. Gionest F. Laprise D. (2013). Spatially explicit structure of natural stands dominated by black spruce. *Silva Fennica* 47:973 10.14214/sf.973

[B36] Rossi S. Simard S. Deslauriers A. Morin H. (2009a). Wood formation in *Abies balsamea* seedlings subjected to artificial defoliation. *Tree Physiol.* 29 551–558. 10.1093/treephys/tpn044 19203970

[B37] Rossi S. Tremblay M.-J. Morin H. Levasseur V. (2009b). Stand structure and dynamics of *Picea mariana* on the northern border of the natural closed boreal forest in Quebec, Canada. *Can. J. For. Res.* 39 2307–2318. 10.1139/X09-152

[B38] Ruel J.-C. Larouche C. Achim A. (2003). Changes in root morphology after precommercial thinning in balsam fir stands. *Can. J. For. Res.* 33 2452–2459. 10.1139/x03-178

[B39] Soucy M. Lussier J. M. Lavoie L. (2012). Long-term effects of thinning on growth and yield of an upland black spruce stand. *Can. J. For. Res.* 42 1669–1677. 10.1139/x2012-107

[B40] Stokes M. L. Smiley T. L. (1968). *An Introduction to Tree-Ring Dating.* Chicago, IL: University of Chicago Press.

[B41] Tremblay M.-J. Rossi S. Morin H. (2011). Growth dynamics of black spruce in stands located between the 51st and 52nd parallels in the boreal forest of Quebec, Canada. *Can. J. For. Res.* 41 1769–1778. 10.1139/x11-094

[B42] Tremblay S. Laflèche V. (2012). *Résultats Obtenus 5 ans Après Traitement dans les Placettes du Réseau de la Mesure des Effets Réels de L’éclaircie Commerciale en Peuplements Résineux*. Peterborough: Ministère des Ressources Naturelles.

[B43] Vincent M. Krause C. Zhang S. (2009). Radial growth response of black spruce roots and stems to commercial thinning in boreal forest. *Forestry* 82 557–571. 10.1093/forestry/cpp025

[B44] Zhang S. Y. Chauret G. Swift E. Duchesne I. (2006). Effects of precommercial thinning on tree growth and lumber quality in a jack pine stand in New Brunswick, Canada. *Can. J. For. Res.* 36 945–952. 10.1139/x05-307

