# Supplementary material for: Does a Spruce Budworm Outbreak Affect the Growth Response of Black Spruce to a Subsequent Thinning?
Source: Front Plant Sci. 2018 Jul 24;9:1061. doi: 10.3389/fpls.2018.01061 (PMC6066515; doi:10.3389/fpls.2018.01061)
Supplement: Supplementary file 1 [file Table_1.DOCX]

**Table S1:** Stands characteristics including the year when thinning was performed. DBH, diameter at breast height. Values are in form of mean ± standard deviation.

| Latitude | Longitude | Altitude  (m a.s.l.) | Number  of trees | Height (m) | DBH (cm) | Minimum  age (years) | Thinning  (year) |
| --- | --- | --- | --- | --- | --- | --- | --- |
| 47°51.3'N | 71°18.2'W | 768 | 37 | 13.3±1.9 | 16.1±3.5 | 126.6±27.3 | - |
| 47°51.8'N | 71°19.1'W | 739 | 37 | 18.2±3.5 | 19.4±4.7 | 68.8±7.6 | - |
| 47°53.2'N | 71°27.8'W | 749 | 37 | 11.5±1.8 | 16.1±3.4 | 59.4±9.6 | 1995 |
| 48°1.9'N | 72°20.0'W | 406 | 31 | 12.9±3.5 | 14.8±4.2 | 73.2±18.6 | - |
| 48°2.0'N | 72°19.8'W | 385 | 24 | 15.1±2.4 | 17.5±2.6 | 84.3±12.4 | 1995 |
| 48°3.2'N | 71°3.8'W | 602 | 35 | 13.3±1.7 | 13.8±2.4 | 109.1±11.9 | 1996 |
| 48°7.6'N | 71°37.4'W | 404 | 35 | 15.3±1.8 | 17.6±2.9 | 63.2±6.9 | 1995 |
| 48°7.8'N | 71°52.8'W | 363 | 30 | 16.4±3.3 | 17.4±4.3 | 63.3±6.4 | 1999 |
| 48°8.3'N | 71°52.8'W | 344 | 35 | 18.4±3.0 | 19.6±3.8 | 68.6±6.6 | 1998 |
| 48°8.4'N | 71°21.6'W | 624 | 40 | 17.1±2.6 | 17.4±3.8 | 66.4±8.6 | - |
| 48°8.6'N | 71°52.7'W | 408 | 20 | 16.8±2.4 | 22.3±3.7 | 63.6±8.6 | - |
| 48°8.6'N | 71°52.7'W | 366 | 39 | 15.7±3.2 | 17.9±4.0 | 66.7±5.2 | 1996 |
| 48°8.6'N | 71°52.5'W | 352 | 34 | 16.5±2.5 | 19.6±4.3 | 63.6±5.4 | 1996 |
| 48°8.7'N | 71°35.3'W | 390 | 43 | 13.7±2.5 | 16.4±5.1 | 61.3±8.9 | - |
| 48°8.9'N | 71°52.3'W | 340 | 31 | 15.2±2.4 | 16.5±3.1 | 64.0±6.5 | 1998 |
| 48°8.9'N | 71°52.0'W | 332 | 78 | 9.3±2.3 | 15.4±2.9 | 62.4±12.9 | 1998 |
| 48°16.7'N | 71°41.2'W | 429 | 30 | 14.7±1.6 | 16.3±2.3 | 65.4±6.6 | 1996 |
| 48°16.7'N | 71°41'W | 397 | 40 | 14.2±2.3 | 17.4±3.3 | 64.2±6.2 | 1996 |
| 48°18.9'N | 71°40.7'W | 314 | 35 | 14.6±2.8 | 21.1±4.1 | 68.7±9.0 | 1996 |
| 48°45.6'N | 70°33.1'W | 673 | 46 | 12.3±1.3 | 15.7±2.3 | 71.2±6.2 | 1996 |
| 48°45.8'N | 70°33.0'W | 676 | 47 | 11.9±2.5 | 14.7±3.8 | 63.4±10.1 | - |
| 48°46.8'N | 70°32.9'W | 658 | 39 | 13.5±2.0 | 16.9±4.4 | 72.1±11.0 | - |
| 48°46.9'N | 70°33.1'W | 652 | 38 | 13.2±1.9 | 18.0±2.7 | 71.4±14.6 | 1998 |
| 48°47.6'N | 70°21.5'W | 662 | 49 | 13.0±1.7 | 17.0±3.0 | 74.7±5.2 | 1997 |
| 48°47.6'N | 70°32.6'W | 618 | 84 | 11.9±1.9 | 16.1±3.3 | 70.4±8.8 | 1995 |
| 48°47.7'N | 70°21.5'W | 662 | 49 | 12.2±1.2 | 12.3±2.3 | 74.7±5.2 | - |
| 48°50.1'N | 71°43.2'W | 202 | 33 | 14.4±2.3 | 14.9±3.4 | 62.7±7.8 | 1997 |
| 48°50.8'N | 70°20.6'W | 589 | 37 | 10.7±1.6 | 11.9±2.2 | 66.5±12.3 | 1998 |
| 48°50.9'N | 70°19.4'W | 605 | 39 | 9.7±1.6 | 11.7±2.2 | 66.9±8.7 | 1997 |
| 48°52.4'N | 71°28.5'W | 201 | 24 | 14.5±2.6 | 15.3±3.6 | 58.5±6.9 | - |
| 48°52.4'N | 71°44.8'W | 245 | 20 | 16.5±2.9 | 20.6±4.9 | 66.4±5.2 | 1997 |
| 48°56.6'N | 71°45.5'W | 231 | 23 | 18.1±3.3 | 20.2±4.2 | 62.6±6.8 | 1999 |
| 48°59.0'N | 72°44.3'W | 214 | 50 | 12.7±1.5 | 14.0±2.6 | 56.0±5.0 | 1996 |
| 48°59.0'N | 72°44.4'W | 221 | 21 | 13.6±1.3 | 13.0±2.6 | 61.9±6.5 | - |
